# Resumes vs. application forms: Why the stubborn reliance on resumes?

**DOI:** 10.3389/fpsyg.2022.884205

**Published:** 2022-07-28

**Authors:** Stephen D. Risavy, Chet Robie, Peter A. Fisher, Sabah Rasheed

**Affiliations:** ^1^Lazaridis School of Business and Economics, Wilfrid Laurier University, Waterloo, ON, Canada; ^2^Ted Rogers School of Management, Toronto Metropolitan University, Toronto, ON, Canada

**Keywords:** applicant screening, application forms, biographical data (biodata) inventories, cover letters, diversity, research-practice gap, resumes, validity

## Abstract

The focus of this Perspective article is on the comparison of two of the most popular initial applicant screening methods: Resumes and application forms. The viewpoint offered is that application forms are superior to resumes during the initial applicant screening stage of selection. This viewpoint is supported in part based on criterion-related validity evidence that favors application forms over resumes. For example, the biographical data (biodata) inventory, which can contain similar questions to those used in application forms, is one of the *most* valid predictors of job performance (if empirically keyed), whereas job experience and years of education, which are often inferred from resumes and cover letters, are two of the *least* valid predictors of job performance (among commonly used screening criteria). In addition to validity evidence, making decisions based on application forms as opposed to resumes is likely to help organizations defend against claims of discriminatory hiring while enhancing their ability to hire in a more diverse, equitable, and inclusive manner. For example, applicant names on resumes can lead to screening bias against members of identifiable subgroups, whereas an applicant’s name can be easily and automatically hidden from decision-makers when reviewing application forms (particularly digital application forms). Despite these convincing arguments focused on applicant quality and diversity, a substantial research–practice gap regarding the use of resumes and cover letters remains.

## Introduction

Most organizations request that applicants provide their resume when applying for job openings (Risavy et al., 2019). However, it is worth asking whether resumes are the most valid method for initially screening applicants and if better alternatives exist that can help to avoid some of the longstanding bias and discrimination issues associated with resume screening. Our perspective is that application forms are a better alternative than the evaluation of resumes and cover letters during the initial screening of applicants. This viewpoint is supported primarily with arguments based on validity and diversity. Overall, this article addresses the extent to which application forms may be more effective than resumes during the initial screening of applicants.

## Initial applicant screening methods

While it is generally understood that resumes and cover letters are documents created by applicants containing what they deem necessary regarding their prior education, work experience, and other information, there are several “classes” of application forms. One such form is the biographical data (biodata) inventory, which asks questions about the personal background and life history of applicants with the intention of using the resulting scores to predict their future behavior (e.g., Mumford et al., 2012). In addition to biodata, there is the weighted application blank (WAB; e.g., Kaak et al., 1998), which asks job-related questions about education and experience and then quantitatively combines the resulting data using a weighted equation. Lastly, if an application form cannot be classified as either biodata or a WAB, then it can be classified as a general application form (i.e., a form comprising job-related questions about education and experience that are qualitatively assessed).

The vast majority of employers in the United States (US; 70.1%) and Canada (82.4%) indicated in a recent study that they analyze resumes and cover letters in their selection process (Risavy et al., 2019). In Germany, the number was even higher: 98.8% (Diekmann and König, 2015). In fact, Risavy et al. (2019) found that analyzing resumes and cover letters was the most common selection tool in the US and Canada after the interview, and Diekmann and König (2015) found that it was the most common selection tool in Germany. Conversely, only a slight majority of employers in the United States (58.4%) and Canada (50.4%) analyze application forms in their selection process (Risavy et al., 2019). In sum, resumes and application forms are two of the most popular initial screening methods for making decisions about applicants, and resumes appear to be more commonly used than application forms, but does the research evidence support the more common use of resumes over application forms?

## Validity evidence

The key factor for organizations to consider when deciding how to initially screen applicants should be validity. Ryan and Tippins (2004) state that, “… it never makes sense to employ a tool without supporting validity evidence—regardless of how cheap, how low the adverse impact, or how easy to administer” (p. 309). Consequently, it is imperative to understand the ability of initial applicant screening methods to predict future job performance (i.e., their criterion-related validity; Tippins et al., 2018).

A recent study that reassessed prior meta-analytic selection tool research with the goal of providing more accurate criterion-related validities found the validity coefficient of empirically keyed (i.e., scientifically–derived) biodata inventories to be 0.38 (corrected for measurement error in the criterion; explaining approximately 14% of the variance in job performance) and the validity coefficient for rationally keyed (i.e., subject matter expert-derived) biodata inventories to be 0.22 (explaining approximately 5% of the variance in job performance; Sackett et al., in press). In fact, the empirically keyed biodata inventory was recognized in this article as the third best predictor of job performance behind the structured employment interview and the job knowledge test (Sackett et al., in press). Similarly, the WAB also appears to have a favorable meta-analytic validity level (Beall, 1991). Despite the potentially robust criterion-related validity associated with the use of biodata inventories, Risavy et al. (2019) found that the biodata inventory was not a commonly adopted selection practice as just 3.9% of United States organizations and 3.4% of Canadian organizations used biodata in their selection process.

Interestingly, there is little information about the criterion-related validity evidence of resumes and cover letters. If we assume that most recruiters are looking for factors such as job experience and years of education when reviewing resumes and cover letters, the validity evidence is weak. The criterion-related validity estimate from Sackett et al. (in press) for job experience (in years) was 0.07 (corrected for measurement error in the criterion; explaining approximately 0.5% of the variance in job performance). There was insufficient recent research for Sackett et al. (in press) to provide an updated validity estimate for years of education, but a previous summary indicated the validity coefficient for years of education to be 0.10 (corrected for both measurement error in the criterion and range restriction; explaining approximately 1% of the variance in job performance; Schmidt and Hunter, 1998). These coefficients likely represent a best-case scenario for resumes and cover letters because it is possible that recruiters are also making judgments based on factors beyond experience and education. For instance, they may use an applicant’s age, which might be inferred from factors such as the date of an applicant’s graduation; age has a –0.01 correlation with job performance (corrected for both measurement error in the criterion and range restriction; Schmidt and Hunter, 1998). Also, the personal statements that are often included in cover letters are likely to have low levels of criterion-related validity and no incremental validity, just as they do in the academic admissions context (Murphy et al., 2009). One possible resume factor that could have reasonably favorable levels of criterion-related validity is grades (Roth et al., 1996), although it is unlikely that grades will be reported in resumes beyond those of recent graduates. A further issue with resumes is the common tendency for applicants to engage in impression management (e.g., Waung et al., 2017) and to misrepresent themselves by providing fabricated or embellished information or by omitting information (e.g., Henle et al., 2019), which would help to explain the lack of validity for this initial applicant screening method.

Based on the criterion-related validity evidence that has accumulated to date, it appears that the biodata inventory has a much higher correlation with job performance than the types of factors that recruiters are likely using to evaluate the resumes and cover letters of their applicants. However, it is possible that the criterion-related validity of a particular biodata inventory may change over time; thus, organizations should continue to collect and analyze data to ensure that the validity of their biodata inventory holds over time. In addition to the favorable criterion-related validity evidence supporting application forms, it is also likely that content validity (i.e., the match between the content of a selection tool and the components of the job) will be improved by using application forms. If the items in an application form are directly related to the qualifications of the job being screened for, then there will likely be high levels of agreement regarding the content validity of this form. In addition to having criterion-related validity and being content valid, it is also beneficial for hiring practices to be unbiased and to help promote diversity, equity, and inclusion. Even in a scenario in which application forms and resumes have similar levels of validity, the need to reduce bias and enhance diversity presents a compelling reason to use application forms over resumes.

## Diversity evidence

It is widely known in the selection literature that structured and standardized selection tools are more valid than unstructured and unstandardized selection tools. For example, there is a significant benefit in terms of criterion-related validity when conducting structured as opposed to unstructured interviews (e.g., McDaniel et al., 1994; Huffcutt et al., 2014). Beyond validity benefits, it is generally accepted that structured interviews are less susceptible to biases such as pre-interview impressions (Macan and Dipboye, 1990) and similar-to-me effects (Sears and Rowe, 2003) than unstructured interviews. These types of biases are important for organizations to avoid as they are likely to be counterproductive for achieving their diversity, equity, and inclusion goals, and can lead to legal liability for discriminatory hiring practices.

During initial applicant screening, using unstructured and unstandardized methods can lead to bias. Applicants choose what information to include in their resumes and cover letters. Thus, there is no structured format across applicants and, as a result, it is unlikely that there will be a consistent manner in which recruiters evaluate resumes and cover letters across applicants. Some applicants may choose to include information pertaining to their extracurricular and volunteer activities; others may choose to include their birthdate, number of dependents, marital status, or even their picture. Consequently, it is unsurprising that decisions made based on resumes and cover letters are rife with discrimination (e.g., Truxillo et al., 2015; Quillian et al., 2017; He and Kang, 2021). Overall, organizations that rely on the evaluation of resumes and cover letters encounter two major issues: (1) there is a lack of comparable data across applicants; and (2) the ability for applicants to include information related to protected grounds then creates the possibility that the organization will be held legally liable for discriminatory hiring practices.

Application forms have a standardized format with fields that are required to be completed by each applicant. This allows for a standardized comparison of data across those applicants. Furthermore, assuming the application form content is job-related and unrelated to protected grounds, recruiters would be unable to make decisions based on protected grounds at this stage of the process and would be better able to successfully defend against claims of discriminatory hiring practices.

As a specific example of an issue regarding bias related to resume reviews, applicants are tempted to engage in “resume whitening” (i.e., the process of applicants changing their name on their resume to avoid discrimination) because of both perceived and real discriminatory decisions against unadjusted resumes, even by employers that are ostensibly in favor of diversity (Kang et al., 2016). One potential solution to resume bias is for organizations to use anonymous application procedures (AAP) that conceal identifying information, such as applicant names (Åslund and Skans, 2012; Derous and Ryan, 2018). The AAP solution has been shown to benefit women and ethnic minorities in terms of interview invitations (Åslund and Skans, 2012); however, even when name-based discrimination can be minimized, there are still other components of the resume that can lead to discrimination, such as implicit age cues (Derous and Decoster, 2017). Furthermore, when HR departments in organizations are responsible for anonymizing resumes, this makes their selection process more cumbersome.

If application forms were used instead of resumes and the initial applicant screening focused solely on job-related minimum qualifications, then this would benefit diverse applicants during initial applicant screening. Moreover, replacing the time-consuming manual resume and cover letter review process with a standardized application form could result in cost savings for organizations because comparing job-related information between applicants would become simpler and the information would be more amenable to anonymization. Overall, in addition to being more valid, it is likely that application forms have an advantage for enhancing diversity, equity, and inclusion in organizations compared with using resumes and cover letters.

## Discussion

Personnel selection is the area of the human resources management literature that suffers from the most severe research–practice gap (Rynes et al., 2002), and this gap appears to be widening (Fisher et al., 2021). Thus, it is perhaps unsurprising that there appears to be a gap between the perspective we advocate here and the most common initial applicant screening method used by organizations. Specifically, although there appears to be research-based validity as well as diversity benefits associated with using application forms over resumes during initial applicant screening, resumes are more commonly used during this stage of the selection process. In order to close this research–practice gap, an important question emerges: why do organizations continue to use resumes instead of application forms in their screening process?

Ryan and Tippins (2004) summarized several possible explanations for the ongoing research–practice gap in selection including the inaccessibility of academic journals, lack of time to revise current practices, and the confusing nature of legal requirements. There are likely many other reasons why practitioners continue using resumes, such as entrenched industry norms and a general resistance to changing their current screening process. The purpose of this article is not only to illuminate research-based insights on the resumes vs. application forms consideration but is also to help practitioners move beyond what appears to be a stubborn reliance on resumes.

The primary recommendation in this article is also one that can likely be implemented by practitioners without a substantial time or cost investment. For example, resources are readily available^1^ to help organizations create application forms that can be used to make standardized decisions based on job-related, minimum qualifications. Moreover, any time or cost investment in moving from resumes to application forms will be recaptured as organizations no longer need to rely on the manual processing of resumes and cover letters. Lastly, although practitioners may be concerned about legal issues, the case we make in this article helps to demonstrate how legal issues can be mitigated when using application forms as opposed to resumes during initial applicant screening. Beyond the practical implications of this article that can hopefully help to begin to close this important research–practice gap, the analysis provided also leads to several other considerations and fruitful research directions.

### Other considerations and future research directions

Future research should seek to understand the underlying reasons why organizations continue to analyze resumes in their screening process as opposed to using application forms and to then use this information to help close this important research–practice gap. One reason why practitioners might not want to adopt application forms could be because of a fear of negative reactions from their applicants; they might assume that busy and qualified professionals would prefer to just submit their standard resume as opposed to being required to complete an organization-specific application form.

Research into applicant reactions has shown that applicant reactions to biodata inventories were ranked near the middle of a set of selection instruments; for example, applicant reactions to biodata were ranked more favorably than personality tests and honesty/integrity tests, but less favorably than work samples and interviews (Anderson et al., 2010). A study by Udechukwu and Manyak (2009) compared applicant reactions to resumes vs. application forms and found that applicants generally perceived application forms to be similar to resumes in terms of several factors (i.e., accuracy, conveyance of information, flexibility, convenience, and preference). The only perception that differed was for ease of use, where resumes were perceived to be easier to use than application forms (Udechukwu and Manyak, 2009). Overall, the research on applicant reactions suggests that fears of negative reactions to application forms are generally unfounded. In fact, a job-related, minimum qualification-focused, and concise application form could be viewed favorably especially in terms of both effort required to complete it and perceptions of organizational decisions, particularly in comparison with a customized cover letter and resume. Nevertheless, future research should further address applicant reactions to resumes and cover letters as well as to different classes of application forms to confirm whether applicant reactions to application forms are more favorable than reactions to resumes and cover letters.

Udechukwu and Manyak (2009) concluded their research on applicant reactions by noting that there may be some value in requesting both a resume and a completed application form; however, the incremental value of the resume in addition to the application form is likely to be negligible and asking for a resume can open the organization to claims of bias and discrimination. Furthermore, it is possible that adding an unstructured predictor (resume) to a structured predictor (application form) will reduce the predictive ability that could have been achieved by solely using a structured predictor (Highhouse, 2008). Regardless, future research should explore whether there is any incremental validity associated with evaluating both resumes and application forms during initial applicant screening.

Beyond resumes and application forms, there are other possible ways to initially screen applicants that we did not address here; for example, recruiters may assess LinkedIn profiles or may meet and interview applicants during job fairs. However, it is unlikely that any of these other initial applicant screening options would be more valid and less biased than using application forms. Regardless, future contributions to the literature should compare other initial applicant screening methods beyond the resume and application form. As organizations rely more on machine learning (Sajjadiani et al., 2019), it will be important for future research to assess whether bias might be better mitigated when the input into algorithms comes from application forms as opposed to resumes.

Lastly, throughout this article we have discussed the potential benefits associated with using application forms instead of resumes during initial applicant screening; however, it would be helpful for future research to formally assess the extent to which organizations realize these benefits. For instance, diversity goals may be realized during initial applicant screening when application forms are used as opposed to resumes and cover letters, but this should be assessed in future research endeavors. Furthermore, assessing whether there are other benefits, such as for employee retention and for time and cost savings, would also be helpful to further add to the body of evidence assessing the extent to which application forms may be more effective than resumes during initial applicant screening.

## Conclusion

All methods used in the selection process must be valid, including those used during initial applicant screening. Beyond validity, all methods used in the selection process should also be unbiased and should help organizations to achieve their diversity, equity, and inclusion goals. Shifting from resumes to application forms can help organizations alleviate bias during initial applicant screening and this shift might just make the world of initial applicant screening a better place for both organizations and their applicants.

## Data availability statement

The original contributions presented in this perspective article are included in the article, further inquiries can be directed to the corresponding author.

## Author contributions

SDR drafted the initial version of the manuscript. CR, PF, and SR made additional, substantial contributions to the conception of the work and to revising it critically for important intellectual content. All authors agree to be accountable for the content of the work.

## Footnotes

1 For example, there are free templates available from the Society for Human Resource Management (SHRM), which can be found by conducting an Internet search using the following: SHRM employment/job application.
